# *Taenia solium* Metacestode Factor as Probable Cause of Temporal Lobe Epilepsy

**DOI:** 10.4269/ajtmh.21-0567

**Published:** 2021-10-18

**Authors:** José L. Molinari

**Affiliations:** Departamento de Bioquímica y Biología Estructural, Instituto de Fisiología Celular, Universidad Nacional Autónoma de México, Ciudad de Mexico, Mexico

## Abstract

This article analyzes data from scientific publications (mainly reviews) concerning the link between human neurocysticercosis and epilepsy. Along with data from our own studies on experimental hippocampal sclerosis induced by a *Taenia crassiceps* metacestode factor in mice, it explores the connection between mechanisms that likely favor the development of epilepsy in cases of human neurocysticercosis. The data from both sources suggest the idea that the *T. solium* metacestode factor causes hippocampal sclerosis and later epilepsy in humans with neurocysticercosis.

## INTRODUCTION

The purpose of this article is to selectively review scientific literature about the link between epilepsy and neurocysticercosis (NCC) and to speculate on the role of the *Taenia solium* metacestode factor (MF) as the potential cause of temporal lobe epilepsy (TLE). Neurocysticercosis is one of the most common risk factors for epilepsy, but its association with drug-resistant epilepsy remains uncertain.^1^ Hippocampal sclerosis (HS) is also the most frequent neuropathologic finding in patients undergoing surgery for intractable TLE.^2^ In a subset of NCC patients, epilepsy seems to be a consequence of HS.^3^ Also, it has been reported elsewhere that patients with HS and NCC have different clinical and neurophysiological manifestations than those with HS alone.^4^ Some authors argue that calcified NCC lesions in patients with mesial temporal lobe epilepsy (MTLE) are merely a coincidental finding. Others speculate there might be a pathogenic relationship between both disorders, and some even suspect that, by acting as an initial precipitating injury (IPI), NCC might cause HS and eventually MTLE-HS.^5^ In a systematic review on NCC-related epilepsy, three different clinical presentations were identified: 1) the cysticercotic lesion was epileptogenic, 2) there was a dual pathology, including the cysticercotic lesion with HS, and 3) the cysticercotic lesion was not related to the epileptogenic focus.^6^ Meanwhile, other authors have reported that the presence of perilesional gliosis contributes to epileptogenicity of calcified neurocysticercotic lesions (CNL) and HS, and the resection of both improves the chances of a seizure-free outcome.^7^

At present, it is uncertain whether there is a cause/effect relationship between these pathologic entities (NCC-epilepsy). It is important to point out that, on this subject, the scientific literature about experimental data with animal models is quite scarce. The facts referred above were obtained from clinical studies (reviews). Therefore, to build a speculative hypothesis on the potential role of NCC in epilepsy, I have collated scientific data from other groups whose ideas are related to the previous findings of my group’s research project.

In patients treated with cysticidal medications, a reduction of 82% in the mean number of brain cysts and a reduction of 95% in the mean frequency of seizures (*P* < 0.001) have been observed.^8^ It has also been argued that, after cysticidal therapy, 83% of all patients who had somatosensory and special sensory seizures showed a significant improvement in the control of seizures.^9^ Recently, another review concluded that the benefits of antiparasitic treatment in parenchymal brain cysticercosis clearly outweigh the risks and provided substantive evidence of the role of NCC as a cause of seizures and epilepsy.^10^

The data presented in the last three references have a common denominator: all cysticercotic patients treated with cysticidal medication and/or surgery had a significant improvement in controlling seizures. This suggests that, by destroying the living cysts, the production and secretion of some substance that induces neuronal damage—clinically expressed as epilepsy/seizures and other neurological signs and symptoms—is inhibited.

In a study of 450 British soldiers returning from India to the United Kingdom, researchers observed that they had developed symptomatic NCC after a mean stay of 4 to 5 years in India. The authors of this study found that metacestodes remain in host tissues in a viable nondegenerate state for variable and prolonged periods of time.^11^ In another study, its author reported the absence of an inflammatory reaction around metacestodes in vesicular stage in CT images of neurocysticercotic children.^12^ Recently, the links among treatment with albendazole, NCC cyst evolution, and seizure outcomes have been further explored in another study. During the first few months after the treatment, the patients in the albendazole group had fewer seizures in comparison to the patients in the placebo group; however, in the long run, those in the placebo group had fewer seizures than the treated group.^13^ The results of these works suggest that, as long as some parasites are alive (i.e., viable cysts), patients may experience seizures, which supports the hypothesis that the parasite produces and secretes some toxic substance that inhibits inflammation around cysts (Dixon/Ridaura) and triggers mechanisms that lead to neuropathology (HS/epilepsy/seizures).

### Pathogenic parasite products.

The idea of parasite substances that damage the hosts is not new. In schistosomiasis, it has been shown that products derived from the parasite suppress thymidine uptake (^3^H) by cultured lymphocytes.^14^ Similarly, a factor released by adult schistosomes, which significantly inhibited lymphocyte proliferation and reduced delayed hypersensitivity in rats infected with *S. mansoni*, has been described.^15^ In human NCC, the absence of an inflammatory reaction around viable cysts^12^ made us think that *T. solium* metacestodes could be secreting a substance (or substances) that was able to inhibit immunological processes. Therefore, we began searching for any substance secreted by *T. solium* metacestodes that had cellular inhibitory features. First, metacestodes (25,000) recently dissected from a naturally cysticercotic pig was left to stand overnight in 500 mL of distilled deionized water (DDW) at 4°C. After several steps of purification, using only the DDW without parasites, we managed to isolate a substance of low molecular weight estimated in < 3,500 Daltons. Next, this substance was tested in vitro on cultured human lymphocytes stimulated with phytohemagglutinin, and results showed a significant depressive effect on the thymidine uptake (^3^H) by these cells.^16^ Follow-up studies in vivo showed that treating mice with this substance reduced the inflammatory reaction around subcutaneously implanted metacestodes and significantly decreased the antibody and cellular responses to metacestode antigens.^17^

### *Taenia crassiceps* MF—mouse model.

To determine if a pathogenic substance from the parasite in *T. solium* cysticercosis was causing certain effects, we designed an animal model following one of the Robert Koch’s postulates.^18^ We used a Sephadex-50G column to isolate fractions of low molecular weight (< 1,300 Daltons) from secretions of *T. crassiceps* metacestodes, which were denominated *T. crassiceps* MF. In a first essay, intraperitoneal (ip) infections with *T. crassiceps* metacestodes or subcutaneous (sc) inoculation of male mice with *T. crassiceps* MF lead to severe disruption and apoptosis of seminiferous tubule cells.^19^^,^^20^ In female mice, *T. crassiceps* MF enhanced ovarian follicle atresia and oocyte degeneration, very much like the ip implantation of *T. crassiceps* metacestodes did.^21^ To corroborate the results obtained in vivo with *T. solium* MF,^17^ we also explored the effect of the *T. crassiceps* MF on spleen cells. Results of this essay showed intense apoptosis of spleen cells either in the white pulp or in the red pulp. Also, the levels of CD4^+^ T cells were significantly lower in both groups of treated mice, compared with control mice. The ex-vivo expression of transforming growth factor (TGF) and factor Foxp3 were significantly higher in experimental cells than in control cells.^22^

Seizures, headaches, and neurological deficits have been reported in human NCC, as well as signs of psychiatric disease (65.8%), cognitive decline (87%), altered memory (25%), and attention deficits (100%).^23^ However, the cause of these associated learning and memory deficits is unknown. To elucidate whether the *T. crassiceps* MF plays a role in the induction of HS, we carried out essays using the *T. crassiceps* MF—mouse model. Results of the first essay of mice implanted ip with *T. crassiceps* metacestodes showed extensive apoptosis in hippocampi cells, whether in the dental gyri or in the hilus and CA1-CA3 regions. In the second essay, mice implanted ip with *T. crassiceps* metacestodes or inoculated with *T. crassiceps* MF showed a significant impairment of performance (learning) in the Barnes maze, as well as extensive apoptosis in all regions of both hippocampi, and an intense deterioration of the adjacent cortex and apoptosis of endothelial cells were confirmed.^24^^,^^25^ Finally, data from the last study using transmission electron microscopy confirmed the HS reported in the previous essays and unveiled other abnormalities in the myelin structure of the CA3 neuron axons, as well as in their internal axonal mitochondria. Apoptosis of endothelial cells, surrounded by large tears in the adjacent nervous tissue and apoptotic astrocytes, was also observed (nonpublished data). All these results strongly support the idea that *T. solium* MF may be the causative agent of HS in human NCC.

### The role of *T. solium* MF as cause of epilepsy.

In human NCC, signs and symptoms appear when the metacestode initiates its degeneration. The previously mentioned report about the return to the United Kingdom of 450 British soldiers after staying about 4 to 5 years in India focuses on the beginning of the clinical picture of NCC in these individuals.^11^ In a different study, the absence of an inflammatory reaction around live metacestodes in image studies of children with NCC was highlighted.^12^ These observations suggest evasion of the immunity by means of virulent factors secreted by the parasite. And our report on the significant depressive activity of *T. solium* MF on the thymidine uptake (^3^H) of human lymphocytes in vitro and the inhibition of inflammation around implanted *T. solium* metacestodes in mice supports this idea.^16^^,^^17^ Additionally, *T. crassiceps* MF inoculated sc in mice also induced a significant immunosuppression of spleen CD^+^4 cells.^22^

In human NCC, comorbidities such as HS, epilepsy, or seizures have frequently been observed. However, the cause/effect relationships between these gnoseological entities have not been well cleared up.^26^^,^^27^ Furthermore, the psychiatric symptoms and cognitive deficits observed such as the learning and memory deficits described in patients with NCC^23^^,^^28^ seem to suggest structural and functional damage of the hippocampus. With this in mind, we decided to test whether the *T. crassiceps* MF had a toxic effect on the hippocampal cells of mice. After subjecting implanted and control mice to the Barnes maze, results showed extensive apoptosis of cells in all regions of both hippocampi and significant learning and memory deficits in implanted mice, compared with control mice. These damages and behaviors were observed in mice either implanted ip with *T. crassiceps* metacestodes or with *T. crassiceps* MF.^24^^,^^25^ This HS induced experimentally in an animal model by a substance secreted by *T. crassiceps* metacestodes may be analogous to that observed in patients with NCC produced by *T. solium* MF.

In theory, substances secreted by *T. solium* metacestodes (in cystic stage) and implanted in the CNS, in this case, the *T. solium* MF could infiltrate brain tissue and reach and damage hippocampal cells and their surrounding tissue, including white matter, while the parasite is alive. This process could last a mean of 4 to 5 years, according to Dixon and LImpscomb.^11^ This damage may be cumulative and dependent on the number and localization of the implanted metacestodes. The *T. solium* MF also probably damages the vicinity of viable cysts. In both circumstances, while this process evolves toward cicatrization, structural and biochemical changes may occur in the dead tissues, which may lead to the development of epilepsy in some patients with NCC. Therefore, they may have one or two epileptogenic foci: one being the HS and another being one or more calcified cysts.

Studies that support our hypotheses have reported that the presence of perilesional gliosis contributes to the epileptogenicity of these lesions (HS and calcified cysts), and that the resection of both lesions improves the chances of seizure-free outcomes.^7^ Other study showed that the clinical-topography relationship between seizures and perilesional edema was more frequently observed only in viable cysts in 85% of patients, and this association increased to 95% if perilesional edema was considered as well.^4^

Regarding the epileptogenic calcified cysts, the inflammatory reactions surrounding the parasite that appear early and persist during the metacestode development until it reaches the final granulomatous/calcified stages suggest additional brain injuries on the sites affected by the *T. solium* MF, together with digestive activity of parasite proteases.^29^

In relation to HS in patients with NCC, several researchers have thought about immunopathological mechanisms as causal factors of this pathology. However, such mechanisms have not been elucidated. There are experimental studies on cysticercal substances that are involved in immunological processes, for instance, the *T. solium* glutathione transferase that activates macrophages and favors the development of T helper 1 (Th1)-type response.^30^ These data suggest that when cysticercotic patients are treated with an antihelminthic drug, the destroyed living cysts stop producing *T. solium* MF; therefore the immunosuppression state (Th2) of patients may come to an end, and the resulting granulomatous immune response might help remove the parasites.^31^ The destruction of the living parasites by antihelminthics may also produce the liberation of structural components including the glutathione transferase which may induce an intense Th1 response that in turn would destroy degenerated metacestodes some of which could be epileptogenic foci.

Small noncoding RNA (sRNA) libraries from larvae of *T. solium* and *T. crassiceps* have been constructed. The sequences of these microRNAs strongly downregulate the production of pro-inflammatory cytokines (IFN-γ) and moderately anti-inflammatory cytokines (IL-4) in murine macrophages, which suggest another immunosuppressive mechanism that helps the larvae to establish and remain inside the host.^32^

It has been reported that another E/S product of larval *T. crassiceps* (designed p66) with characteristics of murine IFN-γ acts as an immunomodulator during infection;^33^ the authors of this work suspect that p66 can bind to the IFN-γ receptor and induce apoptosis in T-cells or macrophages. Therefore, after treating cysticercotic patients with antihelminth drugs, immunomodulating substances such as *T. solium* MF, p66, and other parasite E/S would stop being produced.

A relation between hippocampal atrophy and age has been reported in neurocysticercotic patients older than 68 years.^34^ As authors did not define the kind of relation, and considering the longevity of the cyst stage, plus the duration of the following stages before the calcified stage, it is likely that age is an adjuvant factor for the development of hippocampal atrophy in NCC.

Future experimental studies must be carried out to elucidate structures of *T. solium* MF and *T. crassiceps* MF, especially on immunomodulation and immunolocalization (knowing the antigenicity of *T. crassiceps* MF, unpublished data); as well as to determine the presence and concentration of *T. solium* MF in cerebrospinal fluid from neurocysticercotic patients.

## CONCLUSION

*Taenia solium MF* and *T. crassiceps* MF have shown to be toxic and apoptogenic substances that affect several tissues in mice. Also, many authors have suggested a close relationship between human NCC and HS. Since *T. crassiceps* MF inoculated sc in mice produce HS, *T. solium* MF may also produce HS in patients with NCC. Some neurocysticercotic patients who develop HS may experience a certain type of scarring, which in turn causes epilepsy. This scarring also may occur in the vicinity of some degenerated metacestodes.
